# Monoclonal antibodies and aptamers: The future therapeutics for Alzheimer's disease

**DOI:** 10.1016/j.apsb.2024.03.034

**Published:** 2024-04-17

**Authors:** Alvaro Barrera-Ocampo

**Affiliations:** Facultad de Ingeniería, Diseño y Ciencias Aplicadas, Departamento de Ciencias Farmacéuticas y Químicas, Grupo Natura, Universidad Icesi, Cali 760031, Colombia

**Keywords:** A*β*, Monoclonal antibody, DNA aptamers, Cognitive impairment, Alzheimer's disease, Clinical trials, Immunotherapy, Machine learning

## Abstract

Alzheimer's disease (AD) is considered the most common and prevalent form of dementia of adult-onset with characteristic progressive impairment in cognition and memory. The cure for AD has not been found yet and the treatments available until recently were only symptomatic. Regardless of multidisciplinary approaches and efforts made by pharmaceutical companies, it was only in the past two years that new drugs were approved for the treatment of the disease. Amyloid beta (A*β*) immunotherapy is at the core of this therapy, which is one of the most innovative approaches looking to change the course of AD. This technology is based on synthetic peptides or monoclonal antibodies (mAb) to reduce A*β* levels in the brain and slow down the advance of neurodegeneration. Hence, this article reviews the state of the art about AD neuropathogenesis, the traditional pharmacologic treatment, as well as the modern active and passive immunization describing approved drugs, and drug prototypes currently under investigation in different clinical trials. In addition, future perspectives on immunotherapeutic strategies for AD and the rise of the aptamer technology as a non-immunogenic alternative to curb the disease progression are discussed.

## Graphical abstract

Processing of APP to produce A*β* peptides which are the target of sequence-derived monoclonal antibodies currently approved for the treatment of AD.

**Figure:**
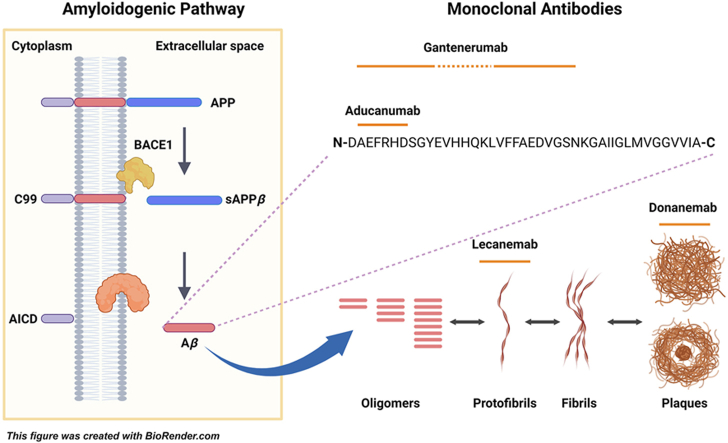

## 1 Introduction

The approval of new anti-A*β* monoclonal antibodies by the FDA (Food and Drug Administration, USA) for the treatment of AD has reignited the interest in immunotherapeutic strategies for this and other neurodegenerative diseases caused mainly by protein misfolding and aggregation. Some years ago, it was reported the failure of many clinical trials involving mAb that showed no cognitive improvement of AD patients despite the observed reduced load of A*β* peptides and the excellent preclinical results^1^. The acceptance by the FDA of aducanumab and lecanemab has been highly controversial among the AD field, but it has opened a window of opportunity for many other antibodies and immune therapies that were stuck in the pipeline for years^2^^,^^3^.

### 1.1 Behavioural and psychological symptoms of AD

Neuroepidemiology studies have established that AD is the most prevalent neurodegenerative illness of adult-onset, with extended prodromal phases characterized by progressive cognitive decline and memory loss. It is also the most common type of dementia accounting for 60%–80% of the cases^4^. It is estimated that the average time course of the disease is 7–10 years, but changes in the brain probably start many years before any clinical symptoms and signs appear. Most of these changes are caused by the death of neurons in regions of the brain responsible for memory processing and other higher cognitive functions.

The entorhinal cortex is one of the first brain regions affected. This structure is connected with the hippocampus and plays a key role in learning and the formation of long-term memories. The neuronal death and decrease in size of these brain areas explain the symptoms observed at the early stages of AD, such as forgetfulness, changes in attention and ability to solve problems. The progression of the disease is characterized by memory decline, language alteration, visuospatial difficulty, loss of awareness and changes in personality, among others. At this stage, the clinical diagnosis is usually made, but further structural damage can be observed in areas of the cerebral cortex responsible for the control of language, conscious thought, reasoning and sensory processing. Changes in the neuronal communication in the cortex can cause delusions, paranoia, hallucinations, anger outbursts and inability to carry out routine tasks (*e*.*g*., eating, bathing, dressing and others). At the end, extensive neuronal damage causes serious atrophy of the cerebral cortex and the enlargement of the ventricles. The patient becomes incapable to recognize its relatives, and cannot control the bladder or bowel function, swallow, walk and sleep^5^.

### 1.2 Risk factors associated with AD

A number of risk factors have been related with the onset of AD. The greatest risk factor for AD is the advanced age, since most of the patients are aged 65 or older^6^. Other risk factors include family history, mild cognitive impairment (MCI), being an apolipoprotein E-*ε*4 (*APOE-ε4*) allele carrier^7^, which associated with the presence of susceptibility genes, such as *ABCA7*, *TREM2* and *SORL1*^8^, may be useful for predicting dementia linked to AD. Additional risk factors include potentially modifiable conditions occurring in midlife, in particular metabolic factors (*e*.*g*., type 2 diabetes, high blood pressure, high cholesterol, obesity, smoking, etc.), and traumatic brain injury^9^, ^10^, ^11^, ^12^, ^13^. In fact, there is increasing evidence that suggests that environmental risk factors, such as exposure to poor quality air, occupational-related exposures and toxic heavy metals, may also contribute to the development of AD^14^.

Increasing amount of evidence has led to the hypothesis that central nervous system infections caused by viruses, bacteria, fungi and parasites may represent an important risk factor because these microorganisms may be able to drive the pathogenesis of AD by inducing neuroinflammation or compromising the integrity of the brain–blood barrier^15^^,^^16^. In this regard, recent multi-omic analysis identified 22 key genes regulated by herpes virus infections, which were associated with the occurrence and development AD^17^. Additional studies have also related infections caused by some bacterial species (*e*.*g*., *Chlamydia pneumonia*, *Porphyromonas gingivalis*, *Borrelia burgdorferi*) with the neuropathogenesis of AD^18^, ^19^, ^20^. However, despite the correlational data presented by these and other studies, a direct relation between the neurodegeneration and the microbial infection is hard to establish because the mechanism underlying the infection and the changes at the cellular and molecular level that could explain the development of AD are largely unknown.

### 1.3 Biomarkers and early diagnosis of AD

The discovery of biomarkers associated with risk factors is an essential step in the early diagnosis, monitoring the onset and the progression of the disease. It is clear that a simple cause of AD is improbable and multiple factors are involved in its progression. In 2018, the NIA-AAA work group proposed that AD could be part of a syndrome formed by multiple diseases characterised by signs and symptoms. However, this definition only makes sense if the person can be accurately diagnosed by its neurocognitive and neuropathologic changes and the use of biomarkers that can support the differential diagnoses to rule out non-AD patients. For this, the NIA-AAA work group outlined an unbiased descriptive classification scheme for biomarkers used in brain aging and AD intended initially for research (interventional trials and observational cohort studies)^5^. They called this classification “AT(N) biomarker grouping”, where A corresponds to aggregated A*β* or associated pathologic state (Cerebrospinal fluid (CSF) A*β*_42_, or A*β*_42_/A*β*_40_ ratio or amyloid PET); T relates to aggregated tau (neurofibrillary tangles-NFTs) or associated pathologic state (CSF phosphorylated tau or tau PET); and N comprehends neurodegeneration or neuronal injury (anatomic MRI, fluorodeoxyglucose PET or CSF total tau)^21^, ^22^, ^23^. It is important to clarify that the biomarkers in the N group are related to neurodegeneration and brain injury as a result of different causes and are not specific for AD. The AT(N) biomarker scheme can incorporate new biomolecules such as TDP43 and *α*-synuclein, and alterations like hippocampal sclerosis, argyrophilic grains and microinfarcts, which can occur alone, or more frequently, along with AD pathologic changes^24^^,^^25^. Thus, the appearance of new therapeutic alternatives makes early and accurate diagnosis essential to increase the likelihood of success in delaying the onset or progress of the disease, which must be reflected in a stabilization of the cognitive decline and an improvement in the quality of life of patients and relatives.

There is no cure for AD and the treatments available until 2021 were only symptomatic. However, the recent approval of a series of mAbs capable of reducing A*β* load in the brain has put the immune therapy at the centre of the stage not only for the treatment of AD, but for other types of dementias. Therefore, this review will discuss the relationship between AD, passive and active immunization with emphasis on the monoclonal antibodies currently available in the pharmacotherapy of the disease and some new molecules that are currently under development and under clinical evaluation.

## 2 Protein aggregation and alternative hypotheses about the pathogenesis of Alzheimer's disease

### 2.1 Neuropathogenesis of Alzheimer's disease

The amyloid cascade hypothesis that embodies the primacy of the biology of amyloid and tau proteins around which much of the drug discovery efforts have been focused in the past 20 years, fails to acknowledge many other covert mechanisms that have been suggested as relevant to elucidate the causes of AD. Understanding the cellular events that lead to neuronal and synaptic loss, which are the likely causes of cognitive impairment in AD, has been a matter of intense research, but many aspects of it remain unknown. In this review, the biology of A*β* is prominently featured as is the base of many mAbs approved or in development as treatment for AD. Nonetheless, it is important to emphasize that there is growing evidence that many other changes occur at the cellular and biochemical level before the appearance of A*β* and tau aggregates^26^, ^27^, ^28^, which has been used for years as markers of AD.

From a neuropathological point of view A*β* and tau aggregates in the form of senile plaques and NFTs are the main hallmarks of AD^29^. The amyloid cascade hypothesis is based in genetic, pathologic, and biochemical evidence that implicate the aggregation of A*β* as a critical trigger in the series of events related to the abnormal deposition in the brain of the hyperphosphorylated tau protein (also known as tauopathy), neuronal dysfunction, death, and dementia^30^. However, the deposition of tau protein correlates with the cognitive decline observed in AD patients^31^, questioning the role of A*β* aggregation as the cause for tau pathogenesis. Analyses of post-mortem human brains revealed a characteristic progression of A*β* deposits and a regular pattern of appearance of NFTs. The progression of A*β* plaques appearance is associated anatomically and functionally with affected brain areas^32^^,^^33^. Extracellular neuritic plaques containing A*β* are found widely distributed throughout the cerebral cortex including the subcortex, allocortex and neocortex while tau-containing NFTs arise first in the medial temporal lobe (entorhinal cortex/limbic brain areas and locus coeruleus), then spread to isocortical regions of the temporal, parietal, and frontal lobes^5^^,^^34^, ^35^, ^36^.

The occurrence of plaques and tangles correlates positively in AD where A*β* deposition occurs in a group of areas known as the default mode network (DMN)^37^, which is distributed in the frontal, temporal and parietal cortex, and the posterior cingulate gyrus. Brain imaging has evidenced that the activity of these regions increases during processes like memory or abstract thought, but decreases during tasks that require attention. Therefore, the DMN is active when the individual is not focused on the external environment^38^^,^^39^. In contrast, tauopathy seems to affect networks that support more specific cognitive domains and are impaired in symptomatic AD, as shown by functional MRI and fluorodeoxyglucose PET^40^, ^41^, ^42^. Recent advances in the elucidation of the brain connectome, which try to determine how billions of neurons are interconnected to form neuronal networks that support cognition and behaviour^43^, have allowed to show that the functional activity in specific brain regions could selectively modify synaptic homeostasis, thus offering an explanation to the convergence of A*β* and tau pathology in AD^43^, ^44^, ^45^. In this regard, it has been suggested that the loss of synaptic homeostasis in areas of high neuronal activity in the DMN could give rise to a cascading network failure affecting the balance in remote but connected brain regions that depend on the DMN for proper functioning^46^. The posterior cingulate cortex is one of these regions highly connected to the medial temporal lobe^47^, therefore, the synaptic stress caused by A*β* accumulation in the posterior cingulate could explain the propensity of the medial temporal lobes to accumulate tau when it loses functional support from the DMN^48^. Thus, during the lifetime of a person prone to developing AD, synaptic dysfunction that leads to A*β* accumulation in the DMN could accelerate tauopathy in strongly linked brain regions and the spreading of tau from the medial temporal lobe to isocortical regions could account for the transition from no symptoms to dementia in AD. However, despite the advances in understanding of the interaction between A*β* and tau, it is still unknown how this interplay affects the organization of the connectome in AD.

### 2.2 Aβ peptides and aggregates

#### 2.2.1 Formation of Aβ

**Figure 1:**
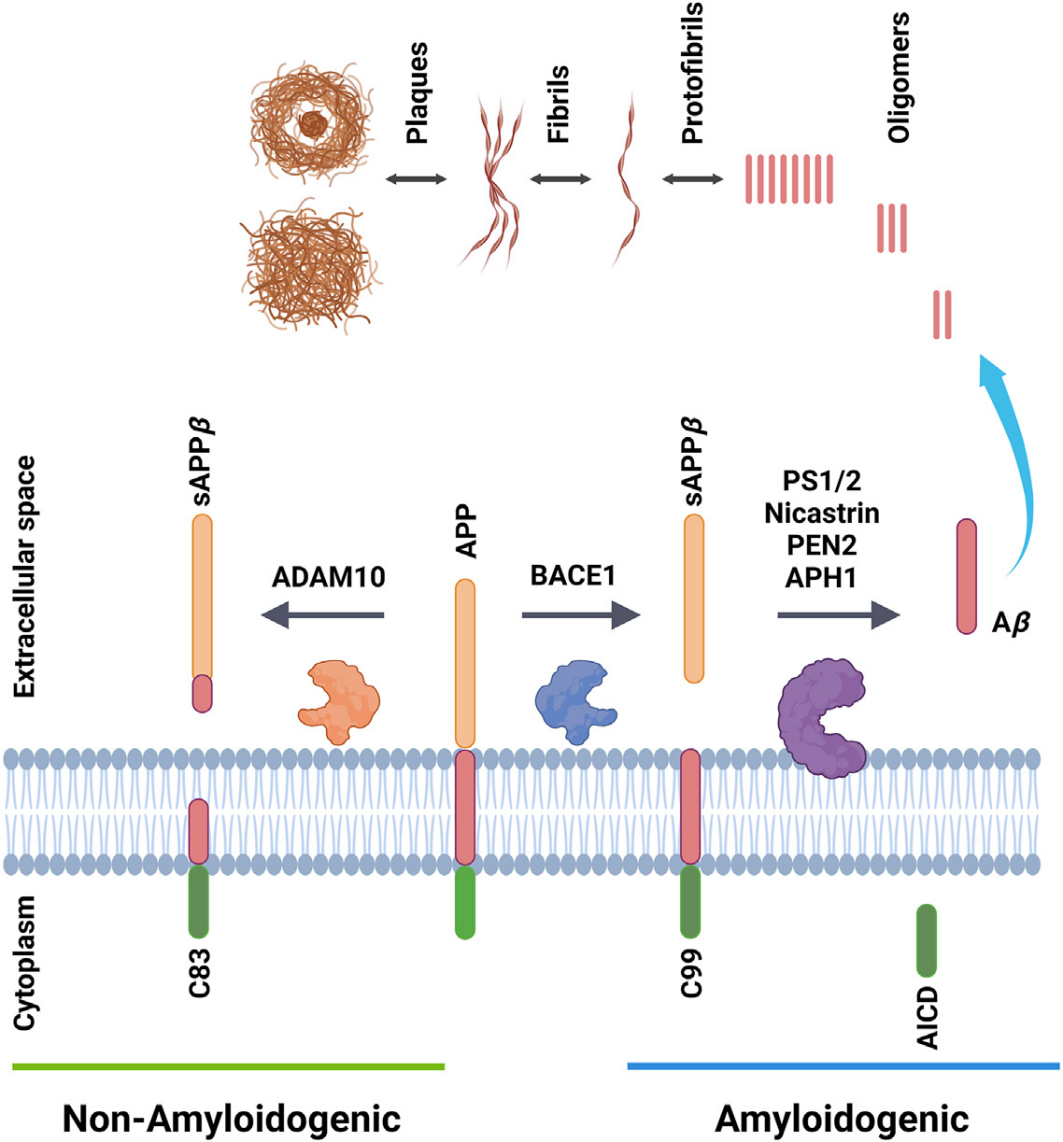
Processing of APP and production of A*β*. APP is initially cleaved by *α*-secretase (ADAM-10) in the Non-amyloidogenic pathway, yielding two fragments: sAPP*α* and C83. The late is cleaved by the *γ*-secretase complex, creating the p3 and AICD peptides. In the Amyloidogenic pathway, *β*-secretase (BACE1) cleaves APP to produce the sAPP*β* and C99 fragments, which are then processed by the *γ*-secretase complex to produce the A*β* and AICD peptides.

The most abundant species of A*β* are 27–43 amino acids in length and are derived from the scission of the amyloid precursor protein (APP), a single transmembrane protein that is enriched in neuronal synapses^49^. APP is produced in neurons and metabolized rapidly^50^. Following the sorting in the endoplasmic reticulum (ER) and Golgi network, APP is translocated to the axon and synaptic terminals. APP processing is made in the trans-Golgi network (TGN) and from there; the protein can be transported to the cell surface or to endosomal compartments through clathrin-associated vesicles. To generate A*β*, APP is endocytosed in clathrin-coated pits into endosomal compartments containing *β*-secretase enzymes. BACE1 is the *β*-secretase enzyme that cleaves the extracellular juxtamembrane region of APP (*β*-cleavage) (Fig. 1). The cleavage of APP by *β*-secretase releases the soluble N-terminus of APP (sAPP*β*) while the C-terminal fragment (CTF-*β* or C_99_) remains bound to the membrane^51^ (Fig. 1). This fragment is subsequently cleaved by *γ*-secretase, which is an aspartyl-type protease membrane protein complex and consists of different several components (Fig. 1). The catalytic elements of the membrane-embedded tetrameric *γ*-secretase complex are represented by presenilin-1 and presenilin-2, which generate the A*β* carboxyl terminus from APP^52^^,^^53^. Nicastrin (NCT), anterior pharynx defective 1 (APH-1) and presenilin enhancer 2 (PEN-2) are additional proteins involved in the *γ*-secretase complex. NCT and APH-1 seem to stabilize the formation of high-molecular-mass protein complex needed for the catalytic activity^54^^,^^55^, whilst PEN-2 is thought to modulate the endoproteolysis of presenilin by forming a stable complex with NCT/APH1^56^. The *γ*-secretase complex interacts with APP in its transmembrane region to create A*β*_40_/A*β*_42_ peptides and the AICD_50_ fragment^57^^,^^58^ (Fig. 1). Once A*β* peptides are produced, they can be released to the extracellular space or degraded in lysosomes. This process is known as the Amyloidogenic pathway^59^, ^60^, ^61^ (Fig. 1).

#### 2.2.2 Aggregation of Aβ

A*β* peptides are found in human brains, both healthy and AD, at subnanomolar concentrations. In the brain tissue various isoforms of A*β* can be found. They differ in the number of amino acid residues at the C-terminal region of the peptide. The most abundant A*β* species in the brain of AD patients is the isoform A*β*_40_, which has 40 residues^62^^,^^63^. Another A*β* species also related to the disease is the isoform A*β*_42_, which is less soluble than A*β*_40_ and it aggregates faster^64^. The structure of A*β* peptides have a characteristic type of *β*-sheet arrangement that favours the aggregation, which leads to the formation of oligomeric species that diffuse through the interstitial fluids. A*β* monomers aggregate and polymerize to form high molecular weight species such as oligomers, protofibrils and fibrils^65^^,^^66^ (Fig. 1). *In vitro* experiments have shown that the aggregation of A*β* starts with a nucleation step, which leads to the formation of disordered oligomers converted afterwards into nuclei that elongate into fibrillar assemblies creating a secondary nucleation process responsible for the proliferation of the aggregates^67^^,^^68^.

#### 2.2.3 Impact of Aβ aggregates on the neuronal function

It is clear now that not only A*β* peptides (A*β*_40_ and A*β*_42_) cause neuronal dysfunction; especially oligomeric forms of the protein seem to be more detrimental to brain functions than the A*β* aggregates such as senile plaques^49^^,^^69^. In this regard, a mixture of dimers and dodecamers was observed in soluble A*β* oligomers derived from human brains^70^; whereas the treatment of primary cultures of hippocampal neurons with A*β* oligomers induced a rapid decrease in membrane expression of memory-related receptors, followed by changes in the morphology of dendritic spines, reduction in spine density, and synaptic deterioration^71^. Moreover, results suggest that soluble A*β* oligomeric species can inhibit electrophysiological activity of hippocampal neurons that may be important for the formation and storage of memory; so, as a consequence, this inhibition seems critical for the development of AD^72^. Another interesting finding suggests that A*β* oligomers also bind to different types of membrane proteins including the *N*-methyl-d-aspartate (NMDA) receptor subunits. The interaction of the oligomers with this receptor inhibits synaptic plasticity and disturbs calcium homeostasis^73^^,^^74^, causing neuronal death. For this reason, targeting A*β* forms can slow the aggregation process and attenuate the cognitive dysfunction that is the main symptom of AD.

### 2.3 Alternative hypotheses about the neuropathogenesis of AD

For more than two decades, hypotheses regarding the amyloid cascade and tau aggregation have dominated the research scene and the development of treatments for AD^75^. This is probably because A*β* and tau are the main histopathological markers of the disease widely found in the diseased population. However, other points of view have emerged in light of new evidence that maintains that the origin of AD is multifactorial. Different studies have shown that a large number of changes occur at the molecular, cellular, and biochemical levels before A*β* and tau aggregates are evident. Thus far, more than 10 hypotheses relating to the pathogenesis of AD have been documented^76^. Some of the most notable are described below.

#### 2.3.1 The cholinergic hypothesis

It was one of the first to try to explain the cause of AD^77^. This postulates that cholinergic system failure is the cause of AD due to the decrease in the activity of the enzyme acetylcholinesterase in regions such as the amygdala, hippocampus, and cortex of AD brains^78^. Studies based on this hypothesis led to the development of the first medications used to treat AD, acetylcholinesterase enzyme inhibitors (AChEIs)^79^, which avoid the degradation of acetylcholine and are useful in the palliative treatment of the disease that enhance quality of life but have no discernible impact on the development or course of AD^80^.

#### 2.3.2 Mitochondrial damage and oxidative stress theories

They suggest that the activity of the mitochondria could affect APP expression, processing, and accumulation of A*β* in AD^81^. The concept is composed of three main parts. First, the mitochondrial function is determined by the genetic background of the person. Second, both genetic and environmental factors may affect the age-related mitochondrial changes. Third, the mitochondrial activity of an individual varies according to the stage or progression of AD^82^, ^83^, ^84^. It has been found in brain tissue from deceased AD patients that there is a decreased number of mitochondria in neurons while oxidative damage is increased. Oxidative stress can be caused by an excess of reactive oxygen species or a weak antioxidant defence^85^. Thus, the imbalance in pro-oxidants and antioxidants is associated with the disruption of redox circuitry and macromolecular damage. In addition, research has found evidence for a direct relationship between oxidative stress and neuronal dysfunction in AD. Both *in vitro* and *in vivo* evidence points to a direct link between neuronal dysfunction and oxidative stress in AD^86^^,^^87^. A*β*-dependent endocytosis is associated with a reduced number of NMDA receptors on the cell surface of neurons. A decreased expression of NMDA receptors can be attributed to neuronal death caused by excitotoxicity, which can be triggered by increased calcium influx, impaired energy metabolism, and mitochondrial damage^88^.

#### 2.3.3 The calcium homeostasis hypothesis

It suggests that A*β* can increase intracellular calcium levels, making neurons more susceptible to environmental stimuli^89^. This hypothesis has been linked to AD and is closely related to learning and memory. A number of studies have demonstrated that A*β* upregulates intracellular calcium in neurons, which disrupts neuronal metabolism, induces neuronal apoptosis, and contributes to memory decline^90^^,^^91^. Additional studies found that calcium overload produce dendrite disintegration, and changes in neuronal morphology depend on the presence and load of A*β*^92^.

#### 2.3.4 The inflammatory hypothesis

There is mounting evidence about the key role of microglia and the adaptative immune response in the neuropathogenesis of AD^93^^,^^94^. Microglial cells are a type of specialized macrophage present in the brain that have been observed near A*β* plaques and NFTs in AD brains^95^^,^^96^. Interestingly, a team of researchers recently used cell cultures and a murine model of AD to establish that the administration of the nanopolyphenol, *α*-mangostin, was capable of reinducing the reactive state of microglia and enhancing the misfolded-protein clearance capacity through shifting glycolysis to oxidative phosphorylation. This confirms the importance of microglia within the neurogenerative process in AD and provides cellular targets for the development of future treatments^97^. Another group using *in vivo* experiments have also shown that glial activation can be induced by inflammatory substances, such as A*β*, which can result in immunological responses and inflammation. Additional studies have observed increased levels of inflammatory cytokines, including, IL-1*β*, IL-12, IL-18, TGF-*β* and TNF-*α*^98^. These molecules could be related to cellular death, and to the development of AD.

A recent work evaluated the relationship between the mutation R136S in the lipid carrier Apolipoprotein E3, denominated Christchurch (APOE3ch), and A*β*-induced tauopathy. For this, the authors used a humanized APOE3ch knock-in mouse crossed to an AD mouse model injected with tau protein from AD brains. Researchers found that the animals had peripheral dyslipidaemia and a significant decrease in plaque-associated tau pathology. In addition, an increase in microglial response and a decrease in amyloid response was observed around A*β* plaques. These results suggest that APOE3ch influences the microglial response to A*β* plaques, and it suppresses tau seeding and spreading induced by A*β*^99^. The findings are also in accordance with a renowned case report that identified a person resistant to autosomal dominant AD caused by a PSEN1-E280A mutation and a homozygous carrier of the APOE3ch mutation^100^. These and other works increasingly emphasize the relationship between lipid metabolism, immune response, inflammation and the development of AD.

## 3 Pharmacotherapy of AD: The old and the new

Two variants of AD have been recognized. The sporadic AD (SAD) variant can be classified as either early- or late-onset (<60 years of age or > 60 years of age) and is characterized by absence of inheritance pattern. In the familial AD (FAD) variant, the disease tends to develop before 60 years of age (early-onset) and the autosomal dominant heritability accounts for ∼1% of the AD cases^101^^,^^102^. Genetic mutations in genes coding for the amyloid precursor protein (*APP*), presenilin-1 (*PS1*) or presenilin-2 (*PS2*) are the main responsible of the FAD variant^103^. The study of FAD cohorts has markedly influenced the understanding of the cellular mechanisms involved in the pathogenesis of AD. Nevertheless, the cause of SAD is still unknown and the aetiology seems to be heterogeneous. The interaction of multiple factors (*e*.*g*., genetic, epigenetic and environment) and the lack of clarity on the molecular and cellular basis of the disease can explain the complexity of this variant.

Regarding the pharmacological treatment of AD, many strategies have been tried so far. These range from traditional palliative approaches to sophisticated disease-modifying strategies such as gene- and immunotherapy. The standard palliative treatments include inhibitors of the enzyme acetylcholinesterase, that inhibit the breakdown of the neurotransmitter acetylcholine (*e*.*g*., rivastigmine, galantamine and donepezil) and are used in mild to moderate AD^104^^,^^105^. Initially, these drugs improve cognition, behavioural symptoms and routine tasks, but they have shown lack of effectiveness and adverse reactions that compromise their use^106^, ^107^, ^108^. Another drug approved for the palliative treatment of AD is memantine, an antagonist of the NMDA receptor, indicated for patients with moderate to severe AD, which presents high drop-out rates attributed to the numerous associated adverse events^104^^,^^105^^,^^109^. The sparse effectiveness of these drugs and the lack of success of the therapeutic strategies tried so far may be attributed to several factors: *i)* the gap in the knowledge about many other genomic, metabolic and biochemical changes that occur years before the onset of the first symptoms of AD^110^, ^111^, ^112^, *ii)* the lack of biomarkers, accurate and precise tools for the early diagnosis and the difficulty to assess the disease stages, and *iii)* the constrains of the drug discovery process. For years there has been an urgent need to find disease-modifying agents and this has opened the doors to new approaches from different areas of biomedicine that seek to generate a quantum leap in the treatment of AD. In this regard, immunotherapeutic strategies emerge with great potential.

### 3.1 Immunotherapy for AD

#### 3.1.1 Active immunotherapy

Accumulation of A*β* is one of the main events related to the neurodegeneration process in AD. Therefore, immunotherapy has become the epicentre of research to avoid A*β* aggregation and promote its clearance from the brain to delay the onset or progress of the disease. This strategy tries to harness the patient's immune system to remove A*β* peptides from the brain preventing the formation of amyloid plaques. This process is called Active A*β-immunotherapy* and it is based on stimulating B cells with synthetic versions of A*β* (fragments of full-length) so that the organism manufactures its own antibodies, which in turn neutralize the A*β* peptides, thus creating a complex that later is cleared out from the brain. Relevant information on some of the most notable A*β* vaccines developed by active immunization is described below.

##### 3.1.1.1 AN1792

The first active vaccine developed for the treatment of AD (https://clinicaltrials.gov; Identifier: NCT00021723) was developed in 2001 by ELAN and Wyeth using the full-length A*β*_42_ peptide (Table 1). The vaccine achieved some positive results including reduced cognitive decline. However, the clinical trial was brought to a halt because ∼6% of the individuals treated with the vaccine developed meningoencephalitis^113^, ^114^, ^115^. Investigations into the cause of this inflammatory process indicated that one of the excipients used in the preparation of the vaccine produced the exposition of the A*β* C-terminal region, which seems to activate the response of T-helper 2 cells^115^. This information was essential for the development of the new generation of vaccines that did not include this region of the peptide.

**Table 1:** Active and passive immunotherapeutic approaches for AD.

Active immunotherapy						
Vaccine	Company of origin	Target	Formulation adjuvant	Clinical trial phase	AD patient status	Result
AN1792	ELAN/Wyeth	A*β*_42_	QS-21, polysorbte 80	IIa-finished	Mild to moderate	No improvement
ACI-24	AC Immune	tetra-palmytoylated A*β*_1–15_ (*β* conformation)	Liposomes	II	Mild	Decreased A*β* levels
CAD106	Novartis	A*β*_1–6_	Bacteriophage Qb protein coat	III	Prodromal	NR
UB-311	United Neuroscience Ltd.	A*β*_1–14_	CpG/Alum	II	Mild	Decreased A*β* levels
**Passive immunotherapy**						
mAb	Company of origin	Antigen or epitope/IgG	Binding species	Clinical trial phase	AD patient status	Result
Aducamab	Biogen	NT A*β*_3-6_ (E)/human IgG1	Oligomers and fibrils	III	Prodromal to mild	Decreased A*β* levels
Lecanemab (BAN-2401)	Biogen/Eisai/BioArctic	A*β*_42_ AM protofibrils (A)/hIgG1	Protofibrils	III	Prodromal	Decreased A*β* levels
Gantenerumab	Roche	NT A*β*_1-10_ and central region A*β*_18–27_ (E)/human IgG1	Monomers, oligomers and fibrils	III	Prodromal to mild	Decreased A*β* levels
Donanemab	Eli Lylli	pGlu3-A*β*/human IgG1	Core plaques	III	Prodromal	Decreased A*β* levels

A: antigen; E: epitope; hIgG: humanized IgG; NT: N-terminal region; CT: C-terminal region; AM: arctic mutation; NR: not reported.

##### 3.1.1.2 ACI-24

In 2009, AC Immune began a phase I/II trial of ACI-24 to evaluate safety, immunogenicity, and efficacy. This vaccine was made using the tetra-palmytoylated A*β*_1–15_ peptide, which favours the *β* sheet folding (Table 1). The design induced the production of antibodies with specificity for the conformation of the peptide and was formulated as liposomes to elicit the immune response^116^. The trial finished in 2018 and ultimately enrolled only 48 patients of which only 36 produced detectable anti-A*β*_42_ antibodies (https://www.clinicaltrialsregister.eu; Identifier: 2008-006257-40). Then in 2018, a phase II trial started testing a new formulation. The results indicate that ACI-24 produced IgM antibody response, but low IgG titers. Levels of A*β*_40_ and A*β*_42_ in CSF were increased compared to the baseline, suggesting target engagement, but no change on amyloid-PET was observed. No central nervous system inflammation or Amyloid-Related Imaging Abnormality (ARIA) cases were reported (https://www.clinicaltrialsregister.eu; Identifier: 2018-000445-39).

##### 3.1.1.3 CAD106

In 2015 Novartis introduced CAD106 (https://clinicaltrials.gov; Identifier: NCT02565511), which underwent clinical trials until 2020. This contained multiple copies the A*β*_1–6_ peptide coupled with a carrier with 180 copies of the bacteriophage QB coat protein for the induction of the immune response (Table 1). There were no meningoencephalitis cases reported during the phase I trials. However, during phase IIa trials one patient developed intracerebral haemorrhage and four individuals presented imaging alterations related with A*β*^117^^,^^118^. The study was terminated due to unexpected changes in cognitive function, brain volume loss, and body weight loss.

##### 3.1.1.4 UB-311

In 2015, United Neuroscience Ltd. started a phase II clinical trial with the vaccine UB-311. The design concept behind UB-311 consisted in the induction of specific activation of Th-1 cells by introducing the A*β*_1–14_ peptide in combination with UBITh® helper T-cell epitope (Table 1). Results of a phase II trial (https://clinicaltrials.gov; Identifier: NCT02551809) indicated that the vaccine had potential to improve the cognitive function in early-to-mild AD patients with 100% responder rate and strong on-target immunogenicity^119^. The clinical trial did not include a placebo group, but compared the increase of AD Assessment Scale-Cognitive Section (ADAS-Cog) scores from baseline in a subgroup of mild AD patients (Mini-Mental State Examination [MMSE] score ≥20) with a moderate AD subgroup. Data show a slower rate of increase in ADAS-Cog in the subgroup of mild AD patients. In addition, the most common adverse events observed with UB-311 were injection site swelling and agitation. The results suggest that UB-311 may have a potential to improve the cognition of patients with early stage of AD^119^. In 2022, the FDA granted UB-311 fast-track designation for AD, facilitating expedited development and review.

#### 3.1.2 Passive immunotherapy

The passive immunotherapy includes the administration of mAbs to target a specific antigen. This technology overcomes the problems of the active immunization taking advantage of three mechanisms that take place once the antibody has crossed the blood–brain barrier^120^^,^^121^. The first mechanism depends on the interaction A*β*–mAb to decrease the formation of toxic aggregates. The second involves the binding between the Fc-*γ* receptors present on the microglia and the Fc domain of the mAb to induce the phagocytosis of the A*β*–mAb complex. In the third mechanism, the formation of the A*β*–mAb complex leads to the activation of the complement-dependent cytotoxicity effect, which causes the lysis of the target cell. A fourth additional mechanism has been described and is based on the interaction of the mAb with A*β* in the peripheral blood to create a concentration gradient that causes the efflux of A*β* from the brain. Next, I present some mAb against A*β* already approved by the FDA or currently on track to approval, which include aducanumab (Aduhelm®), lecanemab (Leqembi®), gantenerumab and donanemab.

##### 3.1.2.1 Aducanumab

**Figure 2:**
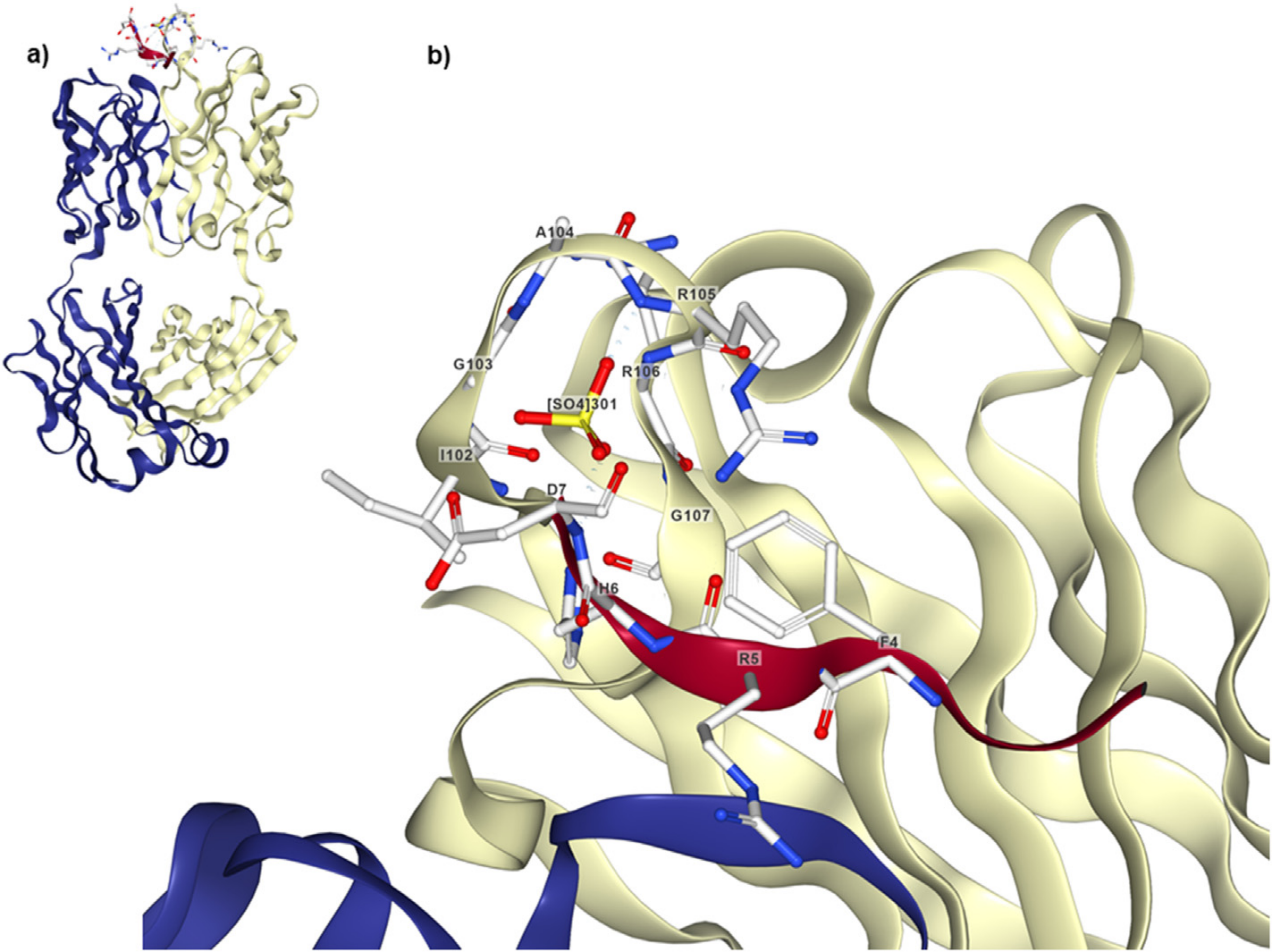
Region of interaction between aducanumab and A*β*. The co–crystal structure reveals the presence of a linear epitope between amino acids 3 and 7 of A*β*. The most essential residues in A*β* to form the complex with the antibody are phenylalanine (F4) and histidine (H6), which are buried inside a hydrophobic pocket formed by the residues tryptophan (W52), tyrosine (Y59), isoleucine (I102), glycine (G103), arginine (R105) and proline (P108) of the heavy chain, and tyrosine (Y32), tyrosine (Y92) and threonine (T94) of the light chain of aducanumab. Thus, explaining the high affinity binding (PDB 6CO3, structured epitope amino acids 3–7)^125^^,^^135^.

It is the first mAb approved by the FDA for the treatment of AD. It was developed by the Swiss biotechnology company, Neurimmune, which in 2007 licensed the development and commercialization to Biogen^122^^,^^123^. This human IgG1 mAb was developed from a library of B cells created from healthy aged individuals^122^. Aducanumab recognizes the A*β* N-terminal region binding to oligomers and fibrils of subjects with prodromal to mild AD (Table 1)^122^^,^^124^. A linear epitope formed by the amino acids 2–7 of A*β* increases aducanumab's affinity towards aggregates of fibrils, as compared to monomers (Fig. 2)^125^. The complex between aducanumab and A*β* is stabilized by the interaction of an arginine on the antibody and a phenylalanine on the peptide, which explains the high affinity binding (Fig. 2)^125^. A preclinical animal model of AD showed that aducanumab reduces brain pathology by stimulating microglia and removing parenchymal, rather than vascular A*β*^122^. In addition, a proteome analysis of the plaque environment found an elevation in proteins involved in metabolism, phagocytosis, regeneration of axons and neurons, as well as reduction in proteins related with A*β* toxicity in a transgenic mouse model of AD treated with aducanumab^126^. In 2012, Biogen started a phase Ib clinical trial (PRIME) (https://clinicaltrials.gov; Identifier: NCT01677572). The randomized, bouble-blind, placebo-controlled study focused on the safety and tolerability of aducanumab and the capability to reduce A*β* load. Results revealed an improvement of cognitive decline in AD patients, but caused ARIA in 41% of the volunteers with high-dose treatment (10 mg/kg). In 2015, before completing the phase Ib study, the company began two phase III placebo-controlled randomized trials (EMERGE and ENGAGE) aiming to compare the clinical efficacy of low or high doses of the antibody with placebo. These trials included volunteers (50–85 years of age) with MCI or mild dementia and PET positive evidence of A*β* pathology in the brain (https://clinicaltrials.gov; Identifier: NCT02477800 and NCT02484547)^127^. Biogen amended the phase III trial protocol based on results of the phase Ib study. This time the dose of aducanumab was increased and the management of the participants who presented mild-to-moderate ARIA was changed by resuming the treatment at the same dose^128^. In 2019, the phase III clinical trials were completed, and the analysis of the results showed that the studies did not have statistically or clinically significant effect. However, the company included additional data and further analyses were made^129^. Then, the results of the two trials were published. The EMERGE trial showed statistically significant effect but clinically doubtful benefit after 78 weeks of treatment according to the Clinical Dementia Rating (CDR) Sum of Boxes score in the subgroup receiving the higher-dose treatment. In contrast, the ENGAGE trial did not show such benefit. Traditionally drug approval depended on 2 rigorous trials showing positive results; however, on June 2021, the FDA gave accelerated approval to aducanumab for MCI and mild AD dementia based on the drug's ability to remove A*β* plaques in the brain^130^. This decision was highly controversial because it was made against the advice of its own advisory committee^131^. Thus far, the relationship between A*β* removal and improvement in clinical outcomes for those with AD is unclear. The accelerated approval requires conducting postmarketing studies to confirm the benefits and in this case, Biogen has 9 years to complete the confirmatory trial, but reliance on future confirmatory evidence is problematic^132^. There are serious concerns about aducanumab's efficacy and adverse effects, which include ARIA and the related symptoms (headaches, confusion, dizziness, nausea, etc.)^133^. Although these effects are worrisome, death is the greatest risk as few fatalities potentially related to aducanumab have been reported^134^.

##### 3.1.2.2 Lecanemab (BAN2401)

It is the second-ever mAb approved by the FDA for the treatment of AD. It was developed by BioArtic and then licensed to Eisai in collaboration with Biogen. This is the humanized version of the murine antibody mAb158, which was raised against APP bearing the Arctic mutation (mutation E22G mutation in A*β*)^136^^,^^137^. The antibody recognizes A*β* protofibrils with much higher affinity than monomers and reduces this specific conformation of the peptide in the brain and CSF of transgenic mice expressing both the Arctic mutation and the “Swedish” double mutation in APP (K670N/M671L; tgArc-Swe mice) (Table 1)^138^. A series of experiments using co-cultures of astrocytes, neurons, and oligodendrocytes from mouse embryos revealed that the treatment with mAb158 can protect neurons from A*β*_42_ toxic concentrations by preventing the accumulation of A*β* through astrocyte-uptake^139^. In addition, recent preclinical studies have shown that the mAb rescues neurons from A*β*-induced cell death, which is associated with improvements in brain perfusion and neuronal viability^139^^,^^140^. In 2010, a phase I clinical trial investigated the safety and tolerability of the antibody. The study was carried out with staggered parallel single and multiple ascending doses, from 0.1 mg/kg as a single dose to 10 mg/kg biweekly for four months. The results proved that the incidence of ARIA-E/H on MRI was comparable to that of placebo and that the antibody was well-tolerated across all doses (https://clinicaltrials.gov; Identifier: NCT01230853)^141^. In 2012, an 18-month phase II proof-of-concept, double-blind study evaluated safety, tolerability, and efficacy of lecanemab in individuals with early AD. This clinical trial assessed three doses across two regimens of lecanemab *versus* placebo, and it showed a 64% probability to be better than placebo. It also determined that the antibody was well tolerated with 9.9% incidence of ARIA-E at 10 mg/kg biweekly. Even though, the study did not meet the 12-month primary endpoint, results from pre-specified 18-month frequentist analyses showed that lecanemab treatment reduced A*β* load and clinical decline by 47% as per ADAS-Cog in a dose-dependent fashion in volunteers with early AD^142^. In addition, the trial established that the optimal dosage for A*β* clearance was 10 mg/kg administered intravenously biweekly, without titration (https://clinicaltrials.gov; Identifier: NCT01767311)^142^. In 2019, it started a phase III study (Clarity AD) to evaluate the efficacy of lecanemab in participants with early AD. The aim was to determine the superiority of lecanemab compared with placebo and to evaluate the long-term safety and tolerability of this in participants with early AD^143^. The clinical trial confirmed the results of the phase II study and met its pre-specified endpoints. After 18 months, the trial showed that lecanemab reduced the burden of A*β* in the brain *versus* placebo and slowed the loss of cognition by 26% according to the ADAS-Cog score. Lecanemab was generally well-tolerated, with a low incidence of ARIA-E (12.6%) and demonstrated a significant slowing of decline in clinical outcomes in early AD (https://clinicaltrials.gov; Identifier: NCT03887455)^143^.

##### 3.1.2.3 Gantenerumab

**Figure 3:**
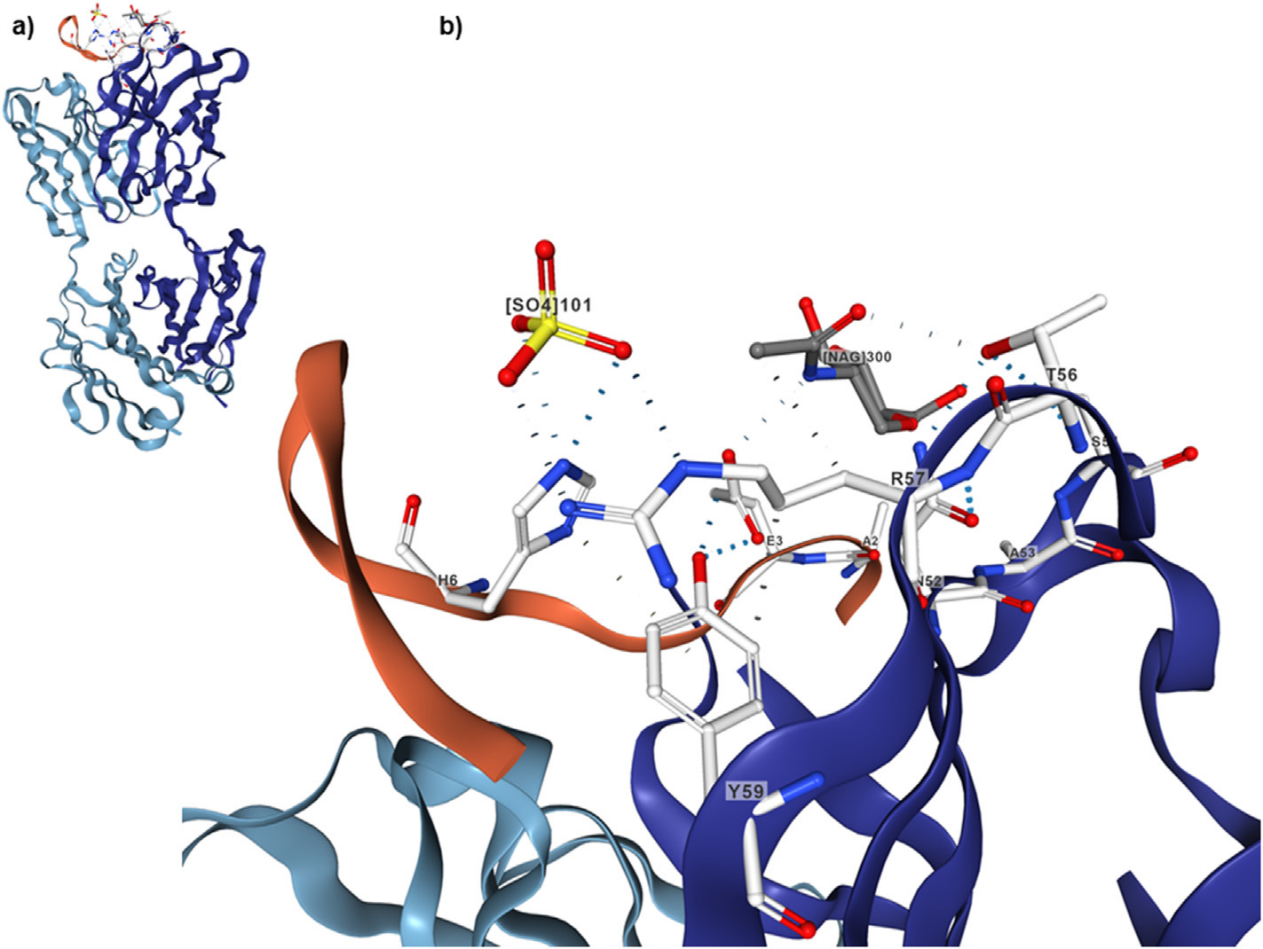
Region of interaction between gantenerumab and A*β*. The co-crystal structure of the antibody and A*β* (PDB 5CSZ) shows a linearized conformation formed by amino acids 1–10 of A*β*. Gantenerumab exhibits nanomolar binding affinity to A*β*_40_ as revealed by the dissociation constants for A*β*_40_ fibrils, oligomers, and monomers: 0.6, 1.2, and 17 nmol/L, respectively^146^^,^^148^. X-ray crystallography of gantenerumab Fab with A*β*_1-10_ monomer reveals that the peptide contacts extensively with histidine (H1), histidine (H2), histidine (H3), and leucine (L1) in the CDR. However, the complex formed with neutral histidine is energetically unstable and the protonation of histidine (H6) seems to control the interaction between the antibody and A*β*^148^.

It was the first fully human mAb designed to bind to a conformational epitope on A*β* fibrils with subnanomolar affinity. The antibody was selected from synthetic human combinatorial antibody libraries created by phage display, followed by *in vitro* affinity maturation using cassette exchanges of complementarity determining regions (CDR)^144^^,^^145^. Experiments of epitope mapping demonstrated that gantenerumab recognizes two discontinuous regions of A*β* (N-terminal and central region), showing the highest affinity at residues 2–11 and 18–27^146^. The complex between gantenerumab and A*β* is characterized by an extended conformation of the peptide in the groove. The first four residues are bound to the heavy chain of the antibody, whereas residues 5 to 11 interact with both heavy and light chains. Two additional residues interact through hydrogen bonds with atoms of the main chain^146^ (Fig. 3). The separate epitopes provide the antibody with the flexibility to bind several sequences and facilitate avidity-enhanced binding on the fibril surface. This flexibility raises the selectivity of the antibody for various A*β* species, and increases the affinity for monomeric, oligomeric, and fibril forms as evidenced by the dissociation constants (monomers: 17 nmol/L, oligomers: 1.2 nmol/L, fibrils: 0.6 nmol/L)^146^. In order to clarify the mechanism throughout gantenerumab elicits the clearance of A*β* from the brain, a phagocytosis assay was performed using brain slices from deceased patients with AD. In this experiment, the slices were treated with the antibody and afterwards they were incubated with primary human macrophages or primary human microglia. Immunohistochemical analysis showed a concentration-dependent decrease of A*β* load mediated by phagocytic activity and further lysosomal degradation^146^. From 2010 to 2016 four phase I clinical trials were conducted to test the safety, tolerability, pharmacokinetics, and pharmacodynamics of gantenerumab in healthy individuals and patients with AD (https://clinicaltrials.gov; Identifier: NCT01224106, NCT01636531, NCT02133937, NCT02882009). One phase I trial evaluated the effect of the antibody on cognition and functioning and the safety and pharmacokinetics in volunteers with prodromal AD (https://clinicaltrials.gov; Identifier: NCT01224106). The volunteers received subcutaneous injections of either gantenerumab or placebo and a subgroup of 16 participants underwent PET scanning to assess brain amyloid. In the group who received a 60 mg dose, there was a mean change of −15.6% from the baseline relative to placebo in the levels of A*β* cortical, whereas in the group who received a 200 mg/kg dose it was −35.7%. Two patients in the last group showed transient and focal areas of inflammation or vasogenic edema on MRI scans at sites with the highest level of amyloid reduction^147^. Through this study it was established that there was a dose-dependent relationship in the reduction of A*β* levels and it laid the foundation for subsequent efficacy studies, where 105 mg/kg to 225 mg/kg doses were selected if ARIA did not appear^147^. The development of a formulation for subcutaneous administration of gantenerumab was one of the most innovative aspects achieved by the creators of the antibody, since the drugs approved for the treatment of AD are administered orally and the vast majority of mAbs are administered intravenously requiring specialized personnel in a hospital environment. This imposes an additional strain on the patient and increases costs to the healthcare system.

In 2010, Hoffmann-La Roche started a multicentre, randomized, double-blind, placebo-controlled phase II study to determine the efficacy and safety of 105 and 225 mg/kg doses of subcutaneous gantenerumab every 4 weeks in participants with prodromal AD over a 2-year period. This trial included patients with gradual decline in memory function, impaired episodic memory on testing, a CDR scale global score of 0.5, a memory score of 0.5 or 1 in the same rating test, and biomarker evidence of AD pathology. Then, in 2012 the study was expanded to a phase II/III trial called SCarlet RoAD, which was looking to establish the effect on cognition and function of patients with prodromal AD under treatment for two years with the option of a two-year extension (https://clinicaltrials.gov; Identifier: NCT01224106). The primary endpoint was the change from baseline to week 104 in CDR Sum of Boxes scores and the change in brain amyloid levels as measured by PET. However, the study was halted early based on an interim futility analysis; dosing was discontinued; and the study was unblinded. No differences were observed between groups in the CDR Sum of Boxes from baseline, and for placebo and gantenerumab (105 and 225 mg/kg doses). The incidence of asymptomatic ARIA increased in a dose- and *APOEε4* genotype-dependent manner^149^. Despite stopping the study, it was possible to establish dose-dependent effects based on clinical and biomarker endpoints, thus suggesting that higher dosing with the antibody was necessary to achieve clinical efficacy. Given the outcomes of the phase I gantenerumab PET substudy showing the safe A*β* removal from the brain, a second phase III study in mild AD dementia was initiated. The Marguerite RoAD was a multicenter, randomized, double-blind, placebo-controlled, parallel-group trial evaluating the efficacy and safety of subcutaneous gantenerumab (105 or 225 mg/kg every 4 weeks) in volunteers with mild AD dementia for 2-years (https://clinicaltrials.gov; Identifier: NCT02051608). However, the interruption of the SCarlet RoAD study because the results of the futility analysis, led to a halt in the recruitment of volunteers for the Marguerite RoAD trial, which together with the SCarlet RoAD study were switched to open-label extension studies, with participants titrated up to 1200 mg/kg gantenerumab. These extension studies lowered brain amyloid by an average of 59 centiloid on florbetapir PET, with half of the 28 participants who reached this timepoint falling below the threshold for A*β* positivity, and the rest on trajectory to do so. About one-third of participants in the extension studies developed ARIA-E and 65% of these cases were asymptomatic^150^^,^^151^.

In 2012, gantenerumab was evaluated in parallel with solanezumab in a phase II/III trial called Dominantly Inherited Alzheimer Network Trials Unit (DIAN-TU). This aimed at preventing dementia in people with an inherited autosomal-dominant mutation in *APP*, *PS1*, or *PS2* (https://clinicaltrials.gov; Identifier: NCT01760005). Unfortunately, the DIAN-TU study did not meet its primary endpoint because none of the antibodies demonstrated a positive effect on cognition when compared to placebo, and the cognitive change in the clinically normal group was negligible. However, a significant reduction on A*β* plaques was observed using the Pittsburgh Compound B PET compared with placebo at 2 and 4 years. Furthermore, the antibody seemed to normalize AD biomarkers^152^.

Later in 2018, the phase III GRADUATE 1 and 2 trials began (https://clinicaltrials.gov; Identifier: NCT03443973). During these studies, participants with prodromal or mild AD received up to 1020 mg subcutaneous gantenerumab, given as 510 mg/kg every two weeks for two years. The GRADUATE trials take into consideration A*β* positivity confirmed by CSF or PET; a gradual and universal dose-titration regimen regardless of *APOEε4* status aiming to reduce A*β* plaque levels while minimizing ARIA-E events^153^. Then, in 2021 the study was expanded expecting to reclute 2032 participants until 2024. The volunteers would receive 510 mg/kg of gantenerumab every two weeks. However, on November 2022, Roche (a member of the Genentech Group since 2009) announced that gantenerumab failed to slow cognitive decline on the CDR Sum of Boxes in the GRADUATE trials and informed that it had stopped all gantenerumab studies including recently started phase III SKYLINE trial (https://clinicaltrials.gov; Identifier: NCT05256134). The latter aimed to evaluate gantenerumab in cognitively unimpaired people with CSF or PET evidence of brain amyloid^154^. In spite of that, Roche has already developed a new formulation of gantenerumab based on the “brain shuttle” technology to increase the antibody's ability to enter the brain^155^. This time under the name of Trontinemab (RO7126209), the brain shuttle gantenerumab (RG6102)^156^ is being tested in a phase Ib/IIa trial. This randomized, double blind, placebo-controlled, parallel-group study is aiming to evaluate the safety, tolerability, immunogenicity, pharmacokinetics, and pharmacodynamics of multiple-ascending intravenous doses of the new antibody in volunteers with prodromal or mild to moderate AD with confirmed A*β* plagued based on PET scan (https://clinicaltrials.gov; Identifier: NCT04639050).

##### 3.1.2.4 Donanemab

It is a humanized IgG1 mAb directed against the N-truncated pyroglutamate A*β* peptide at position 3 (pGlu3-A*β*, A*β*pE3). This antibody reduces pGlu3-A*β* by inhibiting the enzyme glutamyl cyclase (QC), thus averting pGlu3-A*β* formation and aggregation in plaques^157^, ^158^, ^159^, ^160^. Pyroglutamate is a cyclic amino acid found at the N-terminal region of some proteins and peptides. pGlu3-A*β* is formed by the cyclisation of either glutamine or glutamate by the enzyme GC at amino acid positions 3 or 11 of A*β*, following truncation by N-terminal proteases^161^. The N-terminal pyroglutamate structure is resistant to degradation by peptidases, thus increasing peptide stability. In addition, pGlu3-A*β* has been found in the hippocampi and cortices of patients with AD in greater concentrations than full-length A*β*, suggesting that it is involved in the early neurodegeneration process^162^. Preclinical data has shown that donanemab binds against either soluble or aggregated conformations of pGlu3-A*β* and it has strong reaction with amyloid plaques, specifically with cored plaques without causing microhemorrhages in mice^160^. Histological analysis using postmortem brain tissue from Down's syndrome or AD patients demonstrated that this antibody is able to recognize a subset of about one-third of A*β* plaques, and strongly reacts with A*β* plaque core. It also binds to vascular A*β* and is able to detect intraneuronal A*β* in a mouse model of AD^163^.

In 2013, Eli Lilly started a phase I study including 100 people A*β* positive (PET scan) with mild cognitive impairment due to AD, or mild AD. The aim was to establish the safety, pharmacokinetics, and pharmacodynamics of five intravenous doses from 0.1 to 10 mg/kg, infused monthly up to four times, and a single subcutaneous injection against placebo (https://clinicaltrials.gov; Identifier: NCT01837641). Only the six participants who received the highest dose showed an A*β* plaque load reduction in the brain of 40%; in this group, no ARIA-E was found, but two asymptomatic cases of ARIA-H were identified. Worryingly, the antibody turned out to be strongly immunogenic causing infusion reaction with flushing, rash, chills, dizziness, and fever, and anti-drug antibodies were found in plasma^164^. Then, in 2015, the company began a second phase 1 study in people with prodromal to moderate AD (https://clinicaltrials.gov; Identifier: NCT02624778). The main outcome was to measure the effect of donanemab on A*β* load by using florbetapir PET. In addition, the study was set to determine blood pharmacokinetics of donanemab, and auto-antibodies raised against the molecule. For this, 61 participants were randomized 3:1 to drug or placebo and three dosing regimens were tested, the first consisting of a single dose of 10, 20, or 40 mg/kg, the second of 10 mg/kg every other week for 24 weeks, and the third of 10 or 20 mg/kg every month for 16 months. Results indicate that the antibody reduced A*β* load by an average of 90–100 centiloids after monthly doses of 10 or 20 mg/kg for 16 months. However, about 25% of the participants developed ARIA-E, of which only two cases presented symptoms that resolved when dosing was discontinued^165^.

Based on the encouraging results from previous studies, Elli Lilly started in 2017 a series of phase II and III trials called TRAILBLAZER-ALZ. The first of them was a phase II study aimed to evaluate safety, tolerability, and efficacy of an 18-month course of donanemab alone and in combination with the BACE inhibitor LY3202626 produced by the same company (https://clinicaltrials.gov; Identifier: NCT03367403). This enrolled participants who had memory deficits for at least six months and had positive flortaucipir PET scans. In this case, donanemab was dosed at 700 mg/kg monthly for the first three months, then 1400 mg/kg for up to 18 months. The main result was to look for changes in the cognitive performance of the participants according to different rating scales (*e*.*g*., Integrated AD Rating Scale - iADRS, ADAS-Cog13, CDR Sum of Boxes, MMSE, ADCS-iADL), as well as A*β* and tau PET and volumetric MRI. In 2018, the administration of the BACE inhibitor was stopped and early in 2021, the company announced that donanemab was able to slow the cognitive decline on the iADRS by 32% compared to placebo at 18 months. The treatment with the antibody produced an average reduction in A*β* plaque by 84 centiloids, from 108 at baseline and 66% of the participants who received the antibody were A*β* negative by the end of the trial. Additional results showed that the treatment decreased the aggregation rate of NFT in the frontal cortex and other brain areas^166^. Regarding the adverse events, at the end from the 272 participants enrolled in the study, 27% developed ARIA-E, with 6% becoming symptomatic. Treated participants also had more ARIA-H, small brain bleeds, and nausea. The results also indicate that 90% of the patients developed anti-drug antibodies and loss of brain volume measured by MRI probably because of inflammation^166^. By the end of 2020, Eli Lilly began recruiting for TRAILBLAZER-ALZ 2, a phase II study focused on volunteers who had shown progressive and gradual memory decline for at least six months (MMSE with scores from 20 to 28) with A*β* and tau PET scans (https://clinicaltrials.gov; Identifier: NCT04437511). The main objective of the trial was to determine the change in CDR Sum of Boxes after 18 months, whilst the secondary outcomes included cognitive deficit scores (*e*.*g*., MMSE, ADAS-Cog13, iADRS, and ADCS-iADL), disease biomarkers (A*β* and tau by PET), volumetric MRI, as well as pharmacokinetics and anti-donanemab antibodies evaluation. Initially, the study was set to be developed during early 2024, but the company decided to extend the study to a phase III trial with 1800 participants. In this case, the main outcome will be to establish changes in the iADRS based on a disease-progression model.

In August 2021, the phase III TRAILBLAZER-ALZ 3 study started enrolling cognitively normal volunteers aged 50 to 55 with increased levels of phosphorylated tau (ptau217). The main goal of this study is to evaluate the safety and efficacy of donanemab in participants with preclinical AD. Nine monthly infusions of the antibody or placebo will be administered, and the individuals will be monitored every six months. The main goal is to determine the clinical progression based on a score above zero on the CDR for two consecutive evaluations. The trial, set to run until 2027, also aims to establish the steady-state concentration in plasma of the antibody, the incidence of anti-donanemab antibodies, and a number of cognitive tests. It is worth mentioning that the assessments and monitoring of the participants are done remotely using phone or video calls. Drug administration, blood draws and complementary tests will be done in specialized centres (https://clinicaltrials.gov; Identifier: NCT05026866). At the end of 2021, Eli Lilly began TRAILBLAZER-ALZ 4, a phase III study looking to compare donanemab to aducanumab on A*β* plaque clearance in participants with early symptomatic AD. This is an open-label, parallel-group, 2-arm trial aimed to determine the number of participants who reach complete plaque clearance after treatment during every four weeks for 18 months according to florbetapir PET. Preliminary results indicate that in the first six months of treatment donanemab eliminated four times more A*β* plaques than aducanumab, whilst adverse events such as ARIA-E were similar in both groups (https://clinicaltrials.gov; Identifier: NCT05108922). Another phase III study called TRAILBLAZER-ALZ 5, started at the end of 2022. The volunteers will receive monthly infusions of donanemab or placebo, and the change on the iADRS at 18 months will be the primary outcome expected to be reached before 2027 (https://clinicaltrials.gov; Identifier: NCT05508789). Recently, Eli Lilly started the TRAILBLAZER-ALZ 6 trial, which will investigate different donanemab dosing regimens and their effect on the frequency and severity of ARIA-E in adults with early symptomatic AD and explore participant characteristics that might predict risk of ARIA. The study is focused on volunteers aged 60 to 85 with gradual and progressive change in memory function for six months with a MMSE score of 20–28 and A*β* PET scan results consistent with the presence of amyloid pathology (https://clinicaltrials.gov; Identifier: NCT05738486). Based on the results and the evidence collected in previous studies, the company is planning to apply for FDA approval in the next months, this after the application for donanemab's accelerated approval was rejected at the beginning of 2023 citing insufficient safety data at the moment of the submission in 2021. Eli Lilly's antibody slows AD and data suggests that donanemab may work better than similar drugs already in the market. However, there is serious concern about the safety of the patients since the company acknowledges deaths and serious brain side effects.

The TRAILBLAZER-ALZ clinical trials represent a magnificent opportunity to advance knowledge about AD, even if the results are not positive in the end. Donanemab is presented as a therapeutic alternative with high potential to modify the progression of AD and, in an ideal scenario, offers patients and their families the possibility of improving the quality of life by preserving cognitive function and reducing the burden of symptoms associated with the disease, which would have a positive impact on the autonomy and well-being of patients. However, the adverse effects reported by the participants evidence a significant risk that must be considered, taking into account fundamental ethical principles such as respect for autonomy and equity in the distribution of risks and benefits. The presence of ARIA-H, small brain bleeds, and decreased brain volume can have disastrous consequences for the health of the patients, potentially exacerbating the symptoms of the disease or favoring the appearance of new complications. Likewise, the formation of anti-donanemab antibodies could affect the efficacy of the treatment and trigger serious immunological reactions for the individual. Even mild adverse effects such as nausea should be weighed, as they can affect the treatment adherence and quality of life of the patient.

### 3.2 DNA aptamers: An alternative to the immunotherapy for AD

Nucleic acids can assume complex structures to act as scaffolds of molecular interaction and create complexes with a great variety of molecules. This principle was the fundamental basis for the development of aptamers more than 20 years ago^167^^,^^168^. Aptamers are single-stranded oligonucleotides (DNA or RNA), with a length less than 100 base pairs, which have the ability to bind to other molecules with high affinity and specificity. Currently, a large number of aptamers can bind several targets, ranging from inorganic molecules to large protein complexes, and whole cells. Aptamers can be considered nucleotide analogues of antibodies, but with very low immunogenicity and toxicity. In addition, its generation is significantly easier and cheaper than the production of antibodies^169^.

The discovery of the aptamers is carried out through an iterative selection process called SELEX (Systematic Evolution of Ligands by EXponential enrichment), in which libraries containing about 1 × 10^15^ oligonucleotides of random sequence are incubated with the target of interest. The molecules that bind to the target are separated from those that do not bind. Subsequently, those oligonucleotides that recognize the target are amplified to increase the population of interacting molecules. This process is repeated several times until a group of sequences with high affinity for the target of interest is obtained^170^.

These oligonucleotides are a very powerful tool that has multiple advantages because they can be produced in greater quantity than the antibodies while preserving the sequence that encodes them; this translates into a high level of reproducibility at the time of manufacturing them. Because the generation process is chemical and not biological, the risk of contamination with bacteria and viruses that can occur in the production of antibodies disappears, reducing the downstream processing costs^171^. Its small molecular size (<30 kDa) facilitates access to regions that cannot be reached by antibodies^172^. In addition, aptamers can be functionalized and integrated with other technologies to increase their plasma half-life, promote passage through biological barriers or interact with a specific cell type^169^. Advances in aptamer engineering have made it possible that many of the limitations presented by these molecules have been overcome. Some of the improvement strategies include modification and optimization to improve the pharmacokinetic properties and make them resistant to degradation by nucleases, among others^173^.

**Figure 4:**
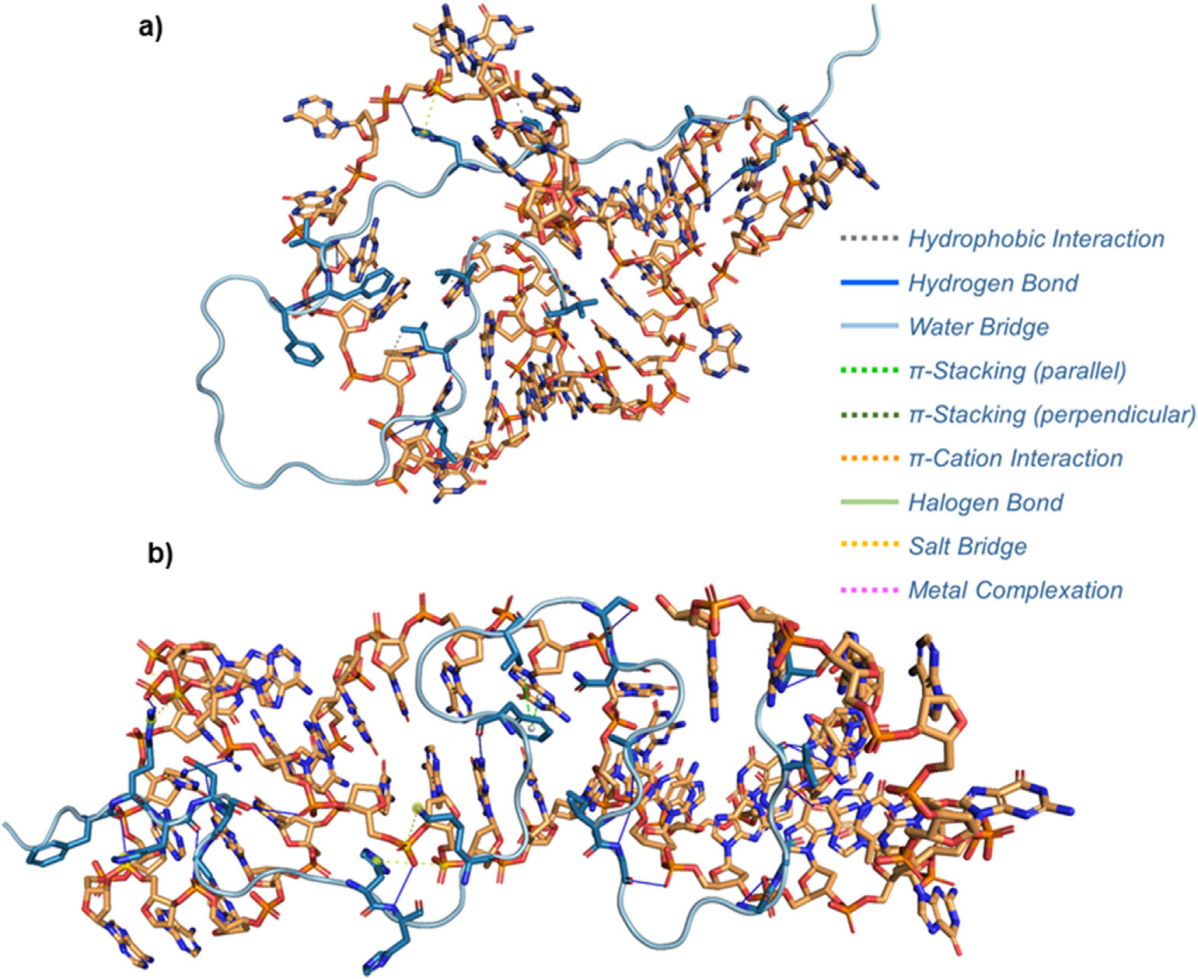
Interaction of the aptamers RNV95 and A*β*7-92-1H1 with A*β*. a) Molecular docking analysis showing the interaction between the A*β*_40_ peptide (light blue) and the RNV95 aptamer (ocher and orange) published by Chakravarthy et al.^175^. In the analysis, it is observed that the most notable interactions between both molecules occur due to the formation of hydrophobic interactions (black dotted lines), salt bridges (yellow dotted lines) and hydrogen bonds (blue lines). b) Molecular docking analysis showing the interaction between the A*β*_42_ peptide (light blue) and the A*β*7-92-1H1 aptamer (ocher and orange) published by Zheng et al.^176^. The analysis reveals that bindings that contribute the most to the interaction between both biomolecules include pi-stacking interactions (green lines), salt bridges (yellow dotted lines) and hydrogen bonds (blue lines). Molecular docking analyses were made using the following software and web platforms: Mfold, RNAfold, RNAComposer, VMD, Chimera X, Yasara, HADDOCK, PLIP, and PDBePISA^177^, ^178^, ^179^, ^180^, ^181^, ^182^, ^183^, ^184^, ^185^.

The therapeutic potential of aptamers to treat neurodegenerative diseases such as AD has begun to be explored. Currently, multiple aptamers have been generated against molecular targets of this disease. In the case of AD, there are aptamers with high affinity for the *β*-secretase enzyme, BACE1; which is involved in the initial step of A*β* production. In this regard, Liang et al.^174^ discovered the A1 aptamer, which inhibits the activity of BACE1 with high specificity and reduces the production of A*β*. Other approaches to this disease are focused on inhibiting the aggregation of A*β* and the high toxicity shown by its oligomers. In 2018, Chakravarthy et al.^175^ by using SELEX developed a number of DNA aptamers with affinity against A*β*_40_. The authors used the ELONA-binding assay and Western blot to establish that the aptamer RNV95 conformed by 39 nucleotides had the capability to bind to tetrameric and/or pentameric A*β* peptide aggregates in neuropathologically confirmed AD brain tissue (Fig. 4a). Then, in 2020, Zheng et al.^176^ generated the A*β*7-92-1H1 aptamer aimed to inhibit the aggregation of both A*β*_42_ monomers and oligomers as demonstrated by atomic force microscopy and surface plasmon resonance analysis. This 44-nucleotide aptamer with a *K*_d_ of 63.4 nmol/L, effectively avoided the formation of amyloid aggregates (Fig. 4b). Altogether, these results show that aptamers have the potential to modify the course of AD by binding with high affinity and selectivity to A*β* species in order to inhibit its aggregation and thus alter/delay the neurodegenerative process of this disease. In addition, these proof-of-concept studies in aptamer technology continue to reveal their promising functionality and immense therapeutic potential. This unique class of biomolecules not only possesses the flexibility of small molecules, but also has the high specificity of antibodies, allowing targeted disease therapy that cannot be achieved with small drug molecules. The particular advantages of aptamers, specially the next-generation aptamer-based therapeutics with superior biological functions and pharmacokinetic profiles are highly anticipated and could fill an important niche market. Furthermore, the technological advancements in synthesis and formulation, and the inclusion of artificial intelligence (AI) and machine learning (ML) in the SELEX process, provide a strong impetus for the development of this promising class of therapeutics.

#### 3.2.1 DNA aptamers: Clinical implications, ethical considerations, and comparison with traditional immunotherapy

DNA aptamers show significant promise in the treatment of neurodegenerative diseases such as AD, but the clinical implications, ethical considerations, and comparative advantages with traditional immunotherapy need to be considered. As this technology evolves, it is essential to ethically and equitably address the challenges and opportunities it presents to ensure its responsible and beneficial use in the clinical setting.

When analysing the clinical implications of aptamers, it is important to take into account aspects such as specificity and selectivity, versatility in design and accelerated development, and lower immunogenic capacity. DNA aptamers can be designed to be highly specific and selective toward their therapeutic targets. This could lead to more precise treatments by avoiding unwanted side effects and minimizing interference with other cellular functions. Likewise, the ability to design specific aptamers for a wide range of targets makes them versatile in the treatment of various neurodegenerative diseases, including AD. This could allow the development of personalized therapies adapted to the individual needs of patients. Aptamer technology enables rapid design and efficient production of therapeutic agents, which could accelerate the development of new treatments and reduce the time to market. Additionally, compared to some traditional treatments, DNA aptamers may have fewer adverse immunological reactions, which could improve the tolerability and safety of the treatment. However, it is essential to carry out exhaustive preclinical studies in order to understand in depth the behaviour and effect of aptamers on the cells and tissues to which they are directed. This is in order to guarantee their safety once they are used on human beings.

Regarding the ethical considerations of using DNA aptamers for the treatment of AD, it is crucial to ensure that patients are fully informed about the nature of aptamers, their potential benefits, and their risks before participating in clinical trials. Transparency in communication and informed consent are fundamental ethical principles. Since aptamers are an emerging technology, patient safety must be prioritized. Continuous monitoring during clinical trials and long-term data collection are essential to evaluate and mitigate any unexpected adverse effects. The issue of equity in access to aptamer-based treatments also needs to be addressed, especially given that scientific advances often face challenges in global distribution and accessibility for different communities.

**Figure 5:**
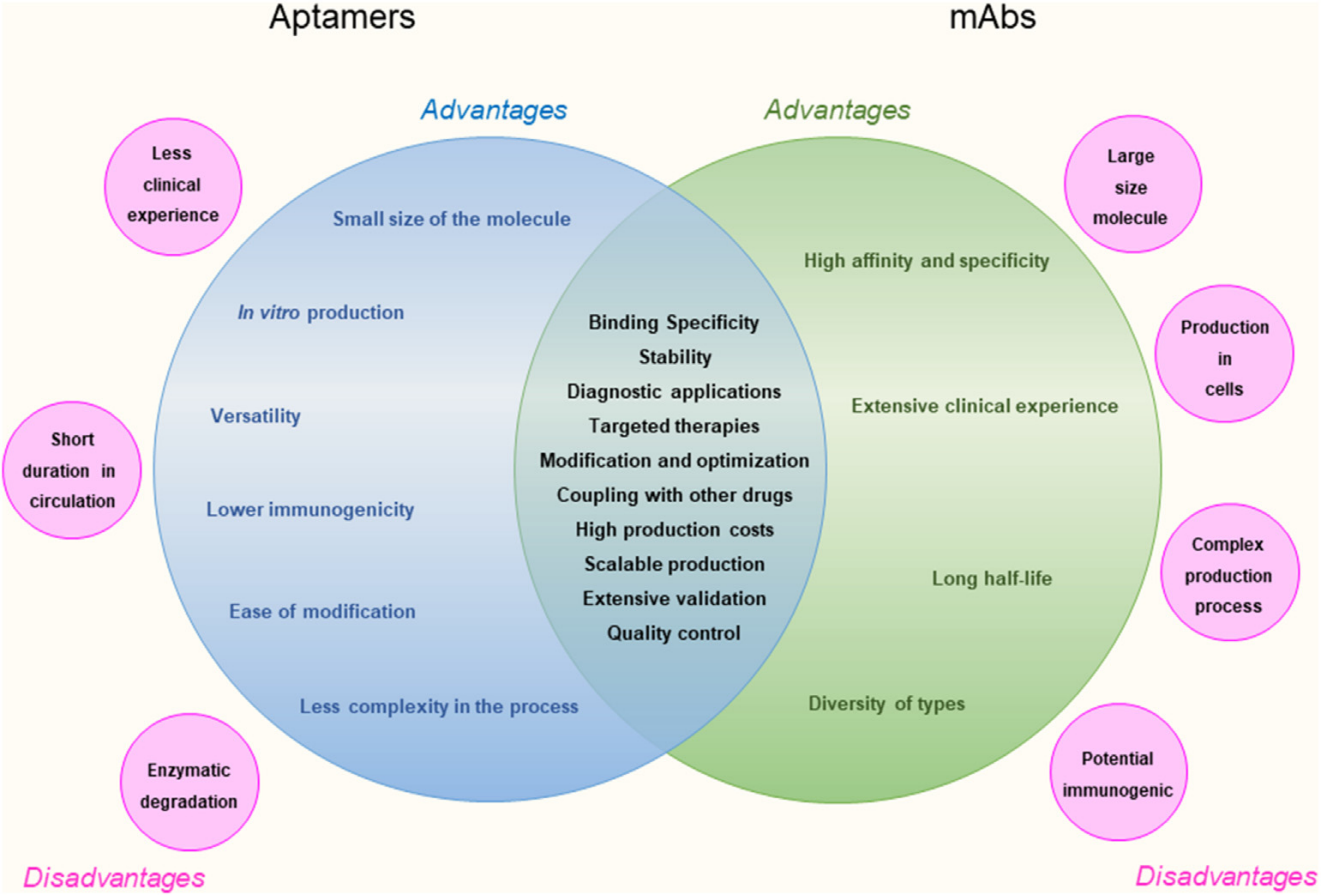
Advantages, disadvantages and similarities between Aptamers and mAbs technology. Each type of technology has advantages, and many of these are similar between the two. However, it is important to highlight that there are also fundamental differences between aptamers and mAbs in terms of structure, production method, and some characteristic properties that may represent a disadvantage when selecting between these types of biomolecules.

When comparing traditional immune therapy and aptamers, it is necessary to analyse in detail critical aspects of each technology, such as specificity and selectivity, structural complexity and development, production and costs, pharmaceutical forms and routes of administration, and immunogenicity (Fig. 5). Regarding specificity and selectivity, both approaches, aptamers and antibodies, can be designed to be highly specific. However, the versatility of aptamers in terms of structural design may allow for greater selectivity for specific targets. On the other hand, the structural complexity of antibodies can be an advantage or disadvantage, depending on the context. While the fixed structure of antibodies can confer stability, the structural flexibility of aptamers allows for more specific and rapid design.

Aptamer production can be more cost-effective and faster compared to antibody production, which often involves the use of live cells in culture. This could influence the accessibility and cost of treatments. Likewise, aptamers can be designed to be administered by various routes, including oral and topical, which could facilitate administration compared to some antibodies that are often administered intravenously. Furthermore, while some antibodies can induce immune responses, aptamers tend to be less immunogenic. This could reduce the risk of adverse reactions from the immune system, thus being safer for patients.

#### 3.2.2 The impact of AI and ML on the development DNA aptamers

The combination of AI and machine learning ML has the potential to transform the development of DNA aptamers for the treatment of neurodegenerative diseases such as AD. These technologies can accelerate the process of aptamer design, optimization, formulation, delivery, and customization, leading to more effective and precise therapeutic approaches to address the complexity of these diseases. In recent years there has been a resurgence in the number of publications related to the application of AI and ML to the discovery of aptamers for various applications, thus demonstrating the enormous potential that exists when combining these technologies.

In 2021, a methodology using ML-guided Particle Display was published. This was used to develop, validate and improve existing experimental candidates, discover new aptamers, and improve therapeutic characteristics. The authors were able to evaluate more than 187.000 aptamers and automatically identify candidates with minimal length and high affinity for the target^186^. More recently, Perez et al.^187^ published a ML algorithm capable of predicting aptamers from conserved primary and secondary structures, taking into account parameters such as sequence abundance, stability, and structure. This algorithm represents an alternative way to select aptamers from extensive libraries bases on patterns, and it could help shorten the discovery of new molecules. However, despite these advances, to date, no work has yet been published that applies AI and ML to the discovery of aptamers directed against Aβ, tau, or other proteins related to neurodegenerative diseases.

The identification and design of specific aptamers for AD-associated proteins such as A*β* and tau can greatly benefit from the power of ML algorithms. AI models can analyse large molecular data sets to predict and optimize aptamer sequences that have improved affinity and specificity for desired therapeutic targets. In this way, the discovery of aptamers can be accelerated, reducing time and cost. Currently, obtaining aptamers using the SELEX method takes around 3 months and has an approximate cost of USD 8000. It is possible that in the short term, through the use of AI and ML, the aptamer selection process could be reduced to weeks or days, and the cost of obtaining them could be less than USD 1000. In this context, it is possible to imagine a scenario where the crystalline structure of monomeric or oligomeric A*β* is presented to the machine. This, in turn, will iterate through thousands of random DNA sequences and the target protein. The result will be candidates with a high probability of success that can subsequently be used to generate aptamers with optimized physicochemical properties. Thus, minimizing the number of experimental trials required for their validation.

The implementation of AI and ML in the discovery of DNA aptamers can also revolutionise the formulation process and the design of pharmaceutical forms of administration. In this case, the algorithms will analyse, for example, the compatibility and stability of the formulation components with the aptamers. Additionally, generative AI could be used to engineer nanometric dosing systems (*e*.*g*., liposomes, dendrimers, polymeric nanoparticles, polymeric micelles, and virus-based nanoparticles) to target the release of the aptamers in tissues or cells of interest. In this way, it would be possible to reduce potential adverse effects in other organs and help establish appropriate doses based on critical pharmacokinetic parameters such as the distribution, metabolism, and elimination of the biomolecule and the selected pharmaceutical form.

In the future, AI and ML could also help personalise patient treatment by analysing clinical, genetic, and molecular data from patients with AD and other neurodegenerative diseases. This would allow aptamers to be tailored to address the unique characteristics of the disease in each individual, improving efficacy and reducing side effects.

## 4 Concluding remarks and future perspectives

Current drug therapy has limited effects on disease progression and often has side effects such as nausea, diarrhoea or dizziness that are bothersome to patients. AD is a complex entity in which a large number of genomic, epigenetic, biochemical and cellular events converge. Therefore, it is unlikely that monotherapy offers any option for a cure, other than slowing the degree and rate of deterioration of brains with the disease.

The era of vaccines and mAbs for the treatment of AD and other neurodegenerative diseases is here to stay, and similar biomolecules are expected to be approved in the coming years, with notable improvements in their pharmacodynamic and pharmacokinetic characteristics. It is very likely that in the next few years we will see mAbs conjugated with other drugs in the market as warheads capable of attacking several targets simultaneously. Nonetheless, despite all the advances made and the new molecules discovered with this technology, the risk of excessive autoimmune or inflammatory reactions, and the interindividual variability in this response could negatively affect patient's health, even causing death. Therefore, to enhance the safety of new therapies, it is paramount to fully understand AD at its core and discover early diagnostic biomarkers that allow targeted and personalized therapy.

Nucleic acid-based compounds such as aptamers offer a viable and interesting alternative to mAbs due to their easy production. Although aptamers exhibit high specificity, their long-term stability and efficacy in a complex biological environment have yet to be fully validated. Furthermore, it is necessary to comprehensively evaluate the risks of this technology, such as the possibility of triggering adverse immunological responses. Likewise, it is essential to address these risks through rigorous preclinical and clinical studies, as well as continuous safety monitoring in the implementation of aptamer-based treatments. This in order to achieve a careful balance between therapeutic efficacy and the management of possible risks to ensure the safety and well-being of patients.

Aptamers offer significant promise in the treatment of neurodegenerative diseases. On the one hand, they present advantages over other technologies thanks to flexibility and optimization in their design and the possibility of producing fewer adverse effects due to their selectivity and specificity. On the other hand, their successful implementation will require carefully addressing challenges such as stability and pharmacokinetic behaviour, the complexity of the biological environment that can expose them to degradation or favour the appearance of adverse immunological reactions, large-scale production, and the clinical validation necessary to demonstrate their safety and efficacy in patients under real disease conditions. Aptamers still have a long way to go in terms of preclinical and clinical studies, but it is possible that in the future these biomolecules will complement the therapeutic arsenal for the treatment of AD and other neurodegenerative diseases.

The future panorama of AD is marked by the combination of advances in early diagnosis, molecular therapies, immunotherapy, and emerging technologies such as AI that are transforming the paradigm of how this disease is addressed. It is necessary to explore the disease in a more effective way through multimodal approaches that generate therapies that not only focus on a single cellular target but on multiple aspects of the pathological process. In this way, more solid and lasting results could be offered. Likewise, the application of AI and Big Data analysis will revolutionize the understanding of the cellular and molecular phenomena that occur in neurodegenerative diseases. Advanced algorithms will be able to analyse large data sets obtained in preclinical and clinical studies and identify complex patterns to assist in early diagnosis, prediction of disease progression, and the development of more effective and safer treatments. This convergence of emerging technologies has the potential to accelerate discoveries and improve our understanding of the mechanisms underlying AD. However, although the future of understanding and treating AD is promising, it is not without challenges. The complexity of the human brain and the variability between individuals pose significant obstacles. Additionally, the need to address ethical issues related to data privacy, informed consent, and equity in access to innovative treatments will become more pressing.

## Acknowledgments

AB-O was funded by Ministerio de Ciencia, Tecnología e Innovación de Colombia (Grants No. CT 775-2018, CT 80740-460-2021 and CT 86980-460-2021, Colombia) and Universidad Icesi – Convocatoria Interna (Grant No. CA041370, Colombia). The author thanks Mateo Fotich (Department of Pharmaceutical and Chemical Sciences, School of Engineering, Design and Applied Sciences, ICESI University. Cali, Colombia) and Victoria Robles (Department of Pharmaceutical and Chemical Sciences, School of Engineering, Design and Applied Sciences, ICESI University, Cali, Colombia) for preparing the figures on the molecular docking of the aptamers.

## Author contributions

Alvaro Barrera-Ocampo carried out the bibliographic review, analysis and interpretation of results necessary for the preparation of the manuscript. Additionally, he wrote the entire document. The author has approved the final article should be true and included in the disclosure.

## Conflicts of interest

The author declares no commercial interest that could represent a conflict of interest.
